# Tumour Necrosis Factor-α Regulates Human Eosinophil Apoptosis via Ligation of TNF-Receptor 1 and Balance between NF-κB and AP-1

**DOI:** 10.1371/journal.pone.0090298

**Published:** 2014-02-28

**Authors:** Hannu Kankaanranta, Pinja Ilmarinen, Xianzhi Zhang, Ian M. Adcock, Aleksi Lahti, Peter J. Barnes, Mark A. Giembycz, Mark A. Lindsay, Eeva Moilanen

**Affiliations:** 1 The Immunopharmacology Research Group, School of Medicine, University of Tampere and Tampere University Hospital, Tampere, Finland; 2 Department of Respiratory Medicine, Seinäjoki Central Hospital, Seinäjoki, Finland, and University of Tampere, Tampere, Finland; 3 Airway Disease Section, Imperial College School of Medicine at the National Heart and Lung Institute, London, United Kingdom; 4 Department of Physiology & Pharmacology, Snyder Institute of Chronic Diseases, Faculty of Medicine, University of Calgary, Calgary, Alberta, Canada; 5 Department of Pharmacy and Pharmacology, University of Bath, Bath, United Kingdom; UAE University, Faculty of Medicine & Health Sciences, United Arab Emirates

## Abstract

Eosinophils play a central role in asthma. The present study was performed to investigate the effect of tumour necrosis factor-α (TNF-α) on longevity of isolated human eosinophils. In contrast to Fas, TNF-α inhibited eosinophil apoptosis as evidenced by a combination of flow cytometry, DNA fragmentation assay and morphological analyses. The effect of TNF-α on eosinophil apoptosis was reversed by a TNF-α neutralising antibody. The anti-apoptotic effect of TNF-α was not due to autocrine release of known survival-prolonging cytokines interleukins 3 and 5 or granulocyte-macrophage-colony-stimulating factor as their neutralisation did not affect the effect of TNF-α. The anti-apoptotic signal was mediated mainly by the TNF-receptor 1. TNF-α induced phosphorylation and degradation of IκB and an increase in NF-κB DNA-binding activity. The survival-prolonging effect of TNF-α was reversed by inhibitors of NF-κB pyrrolidinedithiocarbamate and gliotoxin and by an inhibitor of IκB kinase, BMS-345541. TNF-α induced also an increase in AP-1 DNA-binding activity and the antiapoptotic effect of TNF-α was potentiated by inhibitors of AP-1, SR 11302 and tanshinone IIA and by an inhibitor of c-jun-*N*-terminal kinase, SP600125, which is an upstream kinase activating AP-1. Our results thus suggest that TNF-α delays human eosinophil apoptosis via TNF-receptor 1 and the resulting changes in longevity depend on *yin-yang* balance between activation of NF-κB and AP-1.

## Introduction

Eosinophils have been implicated in exacerbations of asthma [1] and chronic obstructive pulmonary disease (COPD) [2]. The balance between cell maturation and death is of great importance in determining the number of eosinophils in the blood and tissues [2]. Following *in vitro* culture in the absence of cytokines, eosinophils undergo apoptosis or programmed cell death, a process that can be inhibited by a number of cytokines principally interleukin (IL) -3, IL-5, and granulocyte macrophage-colony stimulating factor (GM-CSF) [3], [4]. Apoptosis is characterised by specific biochemical and morphological changes including cell shrinkage, surface blebbing, chromatin condensation and endonuclease-catalysed DNA break down. This is then followed by fragmentation of the eosinophil into discrete apoptotic bodies that are recognised and engulfed by phagocytic cells without inducing inflammatory reaction [3]–[6]. This process is distinct from cell necrosis which is characterised by cell lysis and uncontrolled release of cellular contents that may be harmful to surrounding tissues [3].

Tumour necrosis factor (TNF)-α is a pleiotropic cytokine exerting growth promotion, cytotoxicity, inflammation and immunomodulation [7], [8]. TNF-α has been suggested to play a significant role in many inflammatory diseases [7]. TNF-α has been shown to activate several inflammatory cells, including eosinophils [7], [8]. There is significant literature to support a pathologic role for TNF-α in asthma, especially in severe refractory asthma and COPD [8]. Even though two recent studies with TNF-α inhibitors failed to demonstrate a favourable risk-benefit profile in severe asthma [9] or COPD [10], TNF-α inhibitors are still regarded as potential new medications in asthma and COPD management [8].

The effects of TNF-α at a cellular level are mediated via TNF-α receptors 1 (TNF-R1; Tnfrsf1a) and 2 (TNF-R2; Tnfrsf1b) [7], [11]. The TNF superfamily consists of more than 35 specific ligand-receptor pairs including e.g. Fas, which is a cell surface receptor for Fas Ligand (FasL) [7]. FasL, after binding to its receptor, induces apoptosis in Fas-bearing cells [3], [7]. Whereas dozens of factors are known to promote growth, differentiation or survival, only a few cytokines, including FasL and TNF-α have been found to induce apoptosis. Fas and the TNF-R1 share a cytoplasmic death domain [12] suggesting that the effects transduced by means of one or the other of these surface receptors would have similar characteristics. Human eosinophils have been reported to express Fas receptor and incubation of eosinophils with the agonistic monoclonal anti-Fas antibody results in apoptotic cell death [3]. In contrast, TNF-α has been proposed to prolong human eosinophil survival, possibly via a mechanism including GM-CSF and p38 mitogen-activated protein kinase activation [13], [14]. In several other cell types, TNF-α has been shown to activate nuclear factor-κB (NF-κB), which has been proposed to mediate cell survival [7], [11]. In fact, there is evidence to suggest the activation and involvement of this pathway in eosinophil survival [15]–[17].

Delayed eosinophil apoptosis is considered to be a pathogenic mechanism in eosinophilic diseases [3]–[6]. In fact, eosinophil apoptosis has been shown to be delayed in asthma and upper airways allergic disease [18], [19]. Given the finding that TNF-α levels are up-regulated in severe refractory asthma and COPD [8], [20], it is tempting to speculate that TNF-α might regulate the longevity of eosinophils as a possible pathogenic mechanism. Thus, we have assessed the extent to which TNF-α regulates human eosinophil apoptosis and the mechanism behind its actions.

## Materials and Methods

### Materials

BMS-345541 (N-(1,8-Dimethylimidazo[1,2-a]quinoxalin-4-yl)-1,2-ethanediamine hydrochloride), gliotoxin ((3*R*,5a*S*,6*S*,10a*R*)-2,3,5a,6-Tetrahydro-6-hydroxy-3-(hydroxymethyl)-2-methyl-10*H*-3,10*a*-epidithiopyrazino[1,2-a]indole-1,4-dione), methylgliotoxin and Igepal CA-630 were purchased from the Sigma-Aldrich Co. (St. Louis, MO, USA). Recombinant human TNF-α and IL-5, anti-human TNF R1 (Tnfrsf1a) and R2 (Tnfrsf1b) neutralising antibodies, recombinant human IL-5, anti-human IL-3 and IL-5 neutralising antibodies and mouse IgG_1_ isotype control were obtained from R&D Systems Europe (Abingdon, UK). Other reagents were obtained as follows: ammonium pyrrolidinedithiocarbamate (PDTC) and SR 11302 ((*E*,*E*,*Z*,*E*)-3-Methyl-7-(4-methylphenyl)-9-(2,6,6-trimethyl-1-cyclohexen-1-yl)-2,4,6,8-nonatetraenoic acid) (Tocris Cookson Ltd., Bristol, UK), monoclonal anti-human Fas [CD95] antibody, clone ZB4 (Kamiya Biomedical Co., Tukwila, WA, USA), monoclonal anti-human TNF-α antibody (Genzyme, Cambridge, MA, USA), monoclonal anti-human CD95 Fas antibody, clone CH-11 and IgM isotype control (Immunotech, Marseille, France), monoclonal anti-human GM-CSF monoclonal antibody (Pharmingen, San Diego, CA, USA), IκBα antibody, actin antibody and horseradish-peroxidase conjugated secondary anti-rabbit antibody (Santa-Cruz Biotechnology, Inc., London, UK), IκBα (phospho S32+S36) antibody (Abcam, Cambridge, UK), horseradish peroxidase conjugated goat anti-mouse antibody (Pierce Biotechnology Inc., Rockford, IL, USA), AP-1 and NF-κB consensus oligonucleotides (Promega Corp., Madison, WI, USA), PD98059 (2-(2-Amino-3-methoxyphenyl)-4*H*-1-benzopyran-4-one) (Calbiochem Novabiochem, Nottingham, UK), SP600125 (Anthra[1-9-*cd*]pyrazol-6(2*H*)-one) (Merck Biosciences, Darmstadt, Germany), soluble recombinant human APO-1/Fas ligand (RhFasL) (Alexis Corp., Laüfelfingen, Switzerland) and tanshinone IIA (1,6,6-Trimethyl-6,7,8,9-tetrahydrophenanthro[1,2-b]furan-10,11-dione Dan Shen ketone) (Enzo Life Sciences AG, Lausen, Switzerland). Other reagents were obtained as described elsewhere [18], [21]–[26].

### Eosinophil purification

Blood (100 ml) was obtained from eosinophilic donors. Donors were either healthy, atopic or asthmatic. They were allowed to use their normal medication (i.e. antihistamines, nasal steroid, beta2-agonist or inhaled glucocorticoid as prescribed). This may affect the basal apoptotic rate of eosinophils as shown previously [18], [27] However, patients on high dose inhaled glucocorticoids (≥1000 µg beclomethasone equivalent/d) or on oral steroids were excluded. Also patients with hypereosinophilic syndrome were excluded. Subjects gave a written informed consent to a study protocol approved by the Ethical Committee of Tampere University Hospital (Tampere, Finland). Eosinophils were isolated to >99% purity under sterile conditions as previously reported [18], [21]–[23]. The cells were resuspended at 10^6^ cells/ml and cultured in Dutch modification of RPMI 1640, 10% fetal calf serum, antibiotics and L-glutamine at 37°C with 5% CO_2_.

### Cell culture

Eosinophils were cultured in the absence or presence of TNF-α and the indicated inhibitors. Gliotoxin and methylgliotoxin were dissolved in ethanol. SB203580 (4-[5-(4-Fluorophenyl)-2-[4-(methylsulfonyl)phenyl]-1*H*-imidazol-4-yl]pyridine), PD98059, SP600125, SR11302 and Tanshinone were dissolved in DMSO. Gliotoxin, methylgliotoxin, BMS-345541, SR11302 and tanshinone IIA were added 60 min before and all other inhibitors 30 min before TNF-α. The pre-incubation times were chosen based on the manufacturer's recommendation or existing literature. The neutralizing antibodies were added 15 min before TNF-α. The cells were incubated under sterile conditions for 40 h for apoptosis assays unless otherwise stated. The final concentrations of ethanol and DMSO were 0.2% and 0.5%, respectively, and this had no effect on the rate of apoptosis in eosinophils (data not shown). However, DMSO may affect the extent of the inhibitory effect of TNF-α on eosinophil apoptosis (in the presence of DMSO (0.5%) the inhibitory effect of TNF-α (10 ng/ml) was 12.7+−2.4% (n = 31; p<0.001), whereas in the absence of DMSO the inhibitory effect of TNF-α (10 ng/ml) on eosinophil apoptosis was 26.2+−2.4% (n = 31, p<0.001). However, use of DMSO could not be avoided in the experiments containing pharmacological inhibitors. A similar concentration of ethanol or DMSO was added to the control cultures.

### Determination of eosinophil apoptosis and viability

Eosinophil apoptosis was determined by using the relative DNA fragmentation assay in propidium iodide (PI)-stained cells and flow cytometry (FACScan, Becton Dickinson, San Jose, CA) as previously described [18], [22]. The cells showing decreased relative DNA content were considered to be apoptotic. The results were confirmed by morphological analysis of cells spun onto cytospin slides and stained with May-Grünwald-Giemsa [22], [23]. Eosinophil apoptosis is expressed as percentage of apoptotic cells (number of apoptotic cells/total number of cells x100). Oligonucleosomal DNA fragmentation, a characteristic feature of eosinophil apoptosis, was analysed by agarose gel DNA electrophoresis as previously described [22]. After electrophoresis, gels were visualised by ultraviolet light and photographed. Eosinophil viability was assessed by PI exclusion in isotonic buffer and analysed by flow cytometry as previously described [22]. Cells impermeable to PI were considered viable. Annexin-V binding assay was performed as previously described [23], [28]. The cells displaying positive Annexin-V FITC labelling [FITC+/PI- and FITC+/PI+] were regarded as apoptotic.

### Extraction of cytosolic proteins and Western blot analysis

Eosinophils (1×10^6^/ml) were incubated in RPMI/FCS in the absence and presence of TNF-α (100 ng/ml). The incubations were stopped at the appropriate times (indicated in the text and figure legends) by centrifugation and cytosolic proteins were extracted as previously described [22], [24]. IκB was then identified and quantified by Western blot analysis using specific antibody as previously described [24]. In another set of experiments, the phosphorylation of IκB was analysed by using a phospho-specific IκB antibody. The quantification of the chemiluminescent signal was carried out with the use of FluorChem software version 3.1 (Alpha Innotech Corporation, San Leandro, CA).

### Preparation of nuclear extracts and electrophoretic mobility shift assay

Eosinophils were incubated for the indicated times with TNF-α (100 ng/ml) in the presence or absence of test compounds. Thereafter the cells were rapidly washed with ice-cold PBS and solubilised in 800 µl of hypotonic buffer A [24]. After incubation for 30 min in ice, 80 µl of 10% Igepal CA-630 was added, the cells were vortexed for 30 s and the nuclei were separated by centrifugation at 21.000 *g* for 10 s. Nuclei were then resuspended in buffer C and nuclear extracts were obtained as previously described [24]. Protein content of the nuclear extracts was measured by Coomassie blue method as previously described [24].

Consensus NF-κB probe containing the decameric NF-κB site (underlined) was 5′-AGT TGA GGG GAC TTT CCC AGGC-3′ (sense strand). For AP-1 consensus oligonucleotide 5′-d(CGC TTG ATG AGT CAG CCG GAA)-3′ was used. Specificity was determined by the prior addition of a 50-fold excess of unlabeled competitor consensus or oligonucleotide. Binding reactions (3 µg of nuclear extract) and separation of protein/DNA complexes from DNA probe by electrophoresis were carried out as previously described [24]. The quantification of densities of specific bands was carried out with the use of FluorChem software version 3.1.

### Statistics

Results are expressed as the mean ± standard error of the mean (SEM). Statistical significance was calculated by paired t-test or analysis of variance for repeated measures supported by the Student-Newman-Keuls or Dunnett's test by using Instat software (GraphPad Software, San Diego, CA, USA). Differences were considered significant if p<0.05.

## Results

### Effect of TNF-α on human eosinophil apoptosis

**Figure 1:**
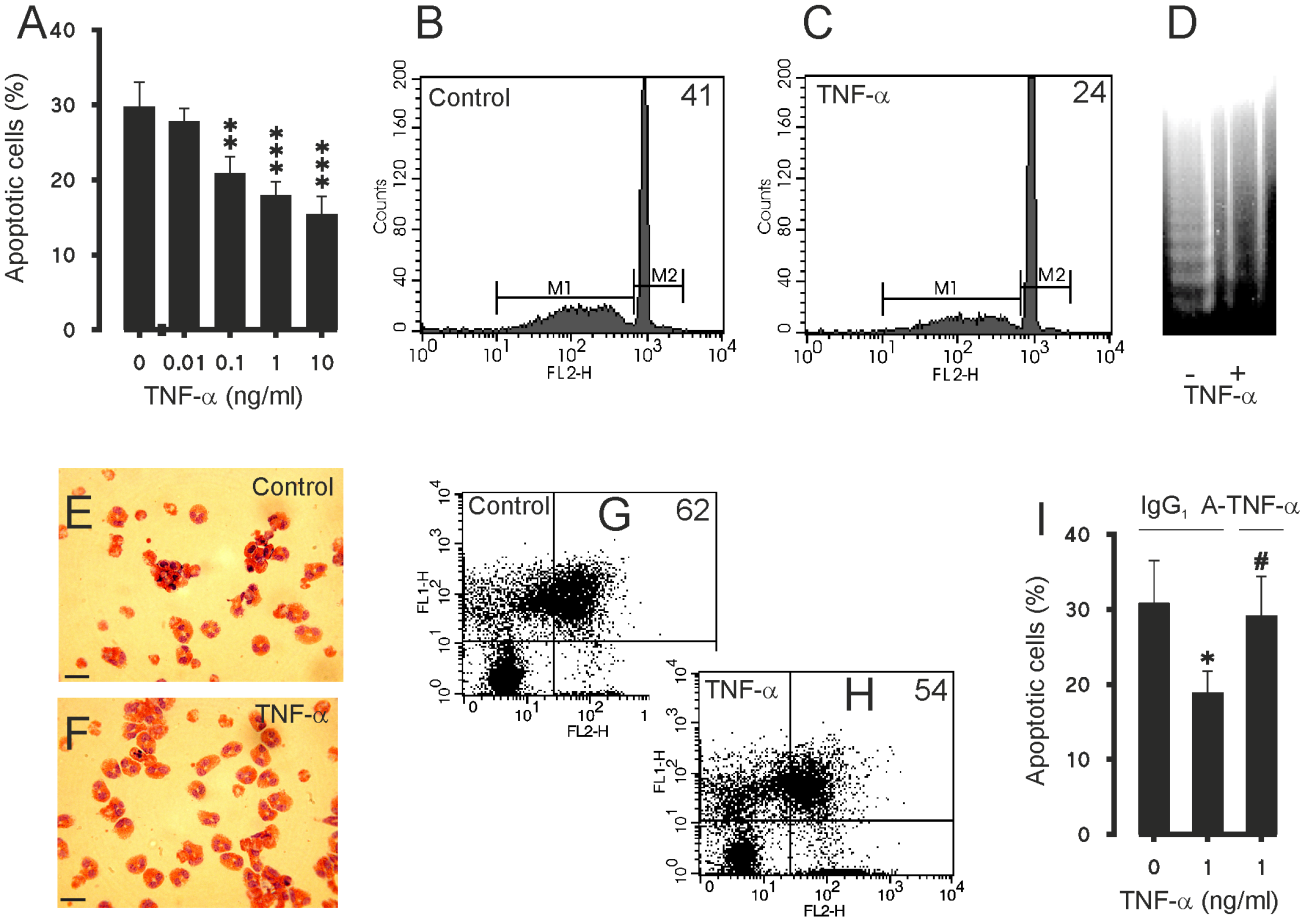
Effect of TNF-α on apoptosis in human eosinophils. Effect of TNF-α on apoptosis in culture for 40 h (A). Representative graphs from relative DNA fragmentation assay of propidium iodide-stained eosinophils are shown in (B and C). In (B and C) figures in top right corner represent percentage of cells showing decreased DNA content. In (D) is shown the DNA fragmentation in eosinophils cultured without (lane 1) and with 10 ng/ml TNF-α (lane 2). The typical apoptotic morphology (cell shrinkage and condensation of nuclear chromatin) of May-Grünwald-Giemsa-stained cytokine-deprived eosinophils is shown in (E) and the reduction in the number of cells showing apoptotic morphology when cultured with TNF-α (10 ng/ml) for 40 h is shown in (F). Scale bar (E and F) is 10 µm. In (G and H) representative graphs from Annexin-V FITC (FL1-H) and uptake of propidium iodide (FL2-H) analysis of eosinophils are shown. In (G and H) figures in top right corner represent percentages of Annexin-V positive cells (FITC+/PI- and FITC+/PI+). In (I) is shown the effect of neutralising TNF-α (A-TNF-α) antibody (5 µg/ml) on the inhibition of apoptosis induced by TNF-α. The isotype control was IgG_1_ (5 µg/ml) and had no effect on eosinophil apoptosis during the 40 h culture. In A and I apoptosis was assessed by flow cytometric measurement of relative DNA content. Each datapoint represents the mean ± SEM of 4–6 independent determinations using eosinophils from different donors. * indicates P<0.05, **P<0.01 and ***P<0.001 as compared with the respective control without TNF-α and # P<0.05 as compared with the respective control without TNF-α neutralising antibody.

TNF-α (0.1–10 ng/ml) inhibited apoptosis in a concentration dependent-manner as determined by flow cytometric analysis measuring the relative DNA fragmentation of PI-stained eosinophils (Fig. 1A–C). The inhibition of apoptosis by TNF-α was also confirmed by the decrease in DNA fragmentation (Fig. 1D) and morphological analysis of May-Grünwald-Giemsa-stained eosinophils (Fig. 1E–F). Culture of eosinophils with TNF-α resulted in a decrease in the number cells showing typical apoptotic morphology in a concentration-dependent manner (Table 1). Furthermore, the inhibition of apoptosis was confirmed by reduced phosphatidylserine expression on the surface of eosinophil as analysed by Annexin V/propidium iodide-counterstaining (Fig. 1 G–H). In the absence of TNF-α 55.3±9.2% of the eosinophils were apoptotic, whereas in the presence of TNF-α (10 ng/ml) 47.3±8.1% of the eosinophils were apoptotic (n = 8, p<0.01). For comparison, in a separate set of experiments, the effect of IL-5 on apoptosis of human eosinophils was analysed by Annexin V-binding assay. TNF-α is clearly less potent inhibitor of apoptosis than IL-5 as there were 10.8±1.8% apoptotic cells in the presence of IL-5 (10 pM) and 62.8±10.0% in its absence (n = 7, p<0.01). To confirm the specificity of the effect of TNF-α, the effects of a neutralising TNF-α antibody were studied. Neutralisation of TNF-α completely reversed the inhibitory action of TNF-α on human eosinophil apoptosis (Fig. 1I). To exclude the possibility that TNF-α would induce primary necrosis (which would lead to a decrease in the number of apoptotic cells), the numbers of primary necrotic cells were determined by PI exclusion. The percentage of primary necrotic cells in control culture after 40 h was 2.8±1.2 (n = 3) and was not affected by the highest concentration (10 ng/ml) of TNF-α (1.9±0.2%, n = 3). Also, to exclude the possibility that the single time point studied (40 h) would explain the result the time-dependency of the effect of TNF-α was studied. TNF-α (100 ng/ml) demonstrated significant suppression of eosinophil apoptosis by 17 h which was maintained and reached a maximum at 40 h (Table 2). Eosinophils have been shown to be able to synthesise TNF-α [29]. To determine whether production of TNF-α by eosinophils themselves would be a trophic factor in non-stimulated eosinophil culture, the effects of the TNF-α neutralising antibody (5 µg/ml) were studied on the rate of spontaneous eosinophil apoptosis. However, neutralisation of TNF-α did not change the rate of spontaneous eosinophil apoptosis (38±10 versus 38±11% apoptotic cells in the absence and presence of TNF-α neutralising antibody after 40 h culture, respectively, n = 4; P>0.05).

**Table 1:** The effect of TNF-α on apoptosis in isolated human eosinophils.

TNF-α (ng/ml)	Percentage of apoptotic cells
0	69±16
0.01	59±20
0.1	44±18
1	32±15*
10	32±5*

Shown is the percentage of apoptotic cells after 40 h incubation as analyzed by morphological criteria. Values are the means ± SEM of four independent determinations using eosinophils from different donors. * indicates P<0.05 as compared with the respective solvent control.

**Table 2:** The time-dependent effect of TNF-α on apoptosis in isolated human eosinophils.

	Percentage of apoptotic cells	
Incubation time	Control	TNF-α (100 ng/ml)
0 h	2±1	2±1
2 h	4±0	6±2
17 h	22±2	14±3*
24 h	37±4	26±5**
40 h	64±5	48±7***

Shown is the percentage of apoptotic cells after 40 h incubation as analysed by relative DNA fragmentation assay. Values are the means ± SEM of four independent determinations using eosinophils from different donors. * indicated P<0.05, **P<0.01 and ***P<0.001 as compared with the corresponding control at the same time point.

### Effect of Fas on human eosinophil apoptosis

**Figure 2:**
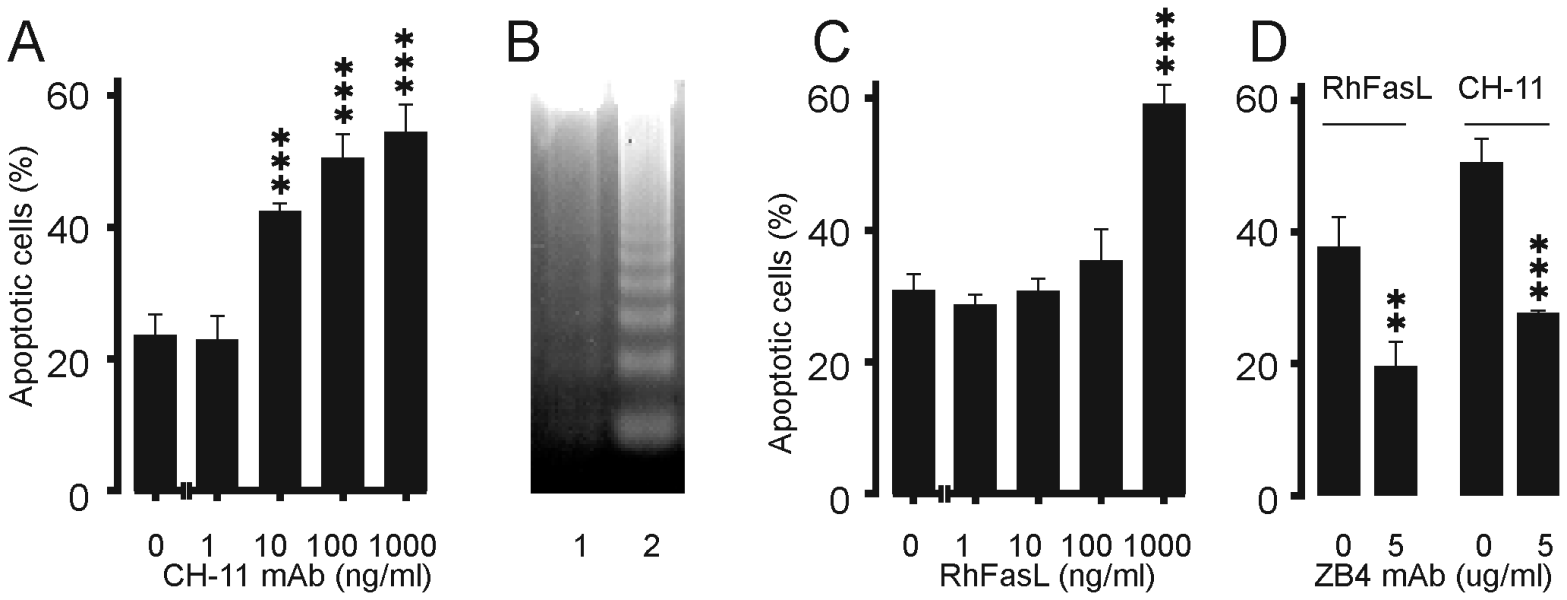
Effect of Fas on apoptosis in human eosinophils. The effect of Fas mAb CH-11 (A) and soluble RhFasL (C) on apoptosis during culture for 40 h. In (B) is shown the effect of isotype control antibody (lane 1; IgM 100 ng/ml) and Fas mAb CH-11 (lane 2; 100 ng/ml) on DNA fragmentation in human eosinophils after culture for 24 h. In (D) is shown the effect of antagonistic Fas mAb ZB4 on human eosinophil apoptosis induced by soluble RhFasL (100 ng/ml) or Fas mAb CH-11 (100 ng/ml). The isotype control for Fas mAb ZB4 was IgG_1_ (5 µg/ml) and it did not affect eosinophil apoptosis. In A, C and D apoptosis was analysed by flow cytometric measurement of relative DNA content. Mean ± SEM, n = 4–5. **indicates P<0.01 and ***P<0.001 as compared with the respective control.

Activation of another member of TNF receptor superfamily, Fas has been previously described to induce apoptosis in human eosinophils [30]–[32]. To confirm that the experimental conditions and methodologies were adequate, the effects of activation of Fas were studied under similar conditions. Similarly to that described previously [30]–[32], cross-linking of CD95 by mAb CH-11 increased apoptosis in human eosinophils (Fig. 2A). Increase in eosinophil apoptosis was also confirmed by increased DNA fragmentation in the agarose gel electrophoresis assay (Fig. 2B). Also the natural ligand of CD95, soluble recombinant human FasLigand (RhFasL) increased apoptotic cell death in human eosinophils (Fig. 2C). The increase in eosinophil apoptosis by mAb CH-11 or soluble FasLigand was also confirmed by morphological analysis of May-Grünwald-Giemsa-stained eosinophils (n = 3, data not shown). The apoptosis-inducing effects of RhFasL and mAb CH-11 were prevented by an antagonistic Fas mAb ZB4, indicating that the effects were mediated by CD95 (Fig. 2D).

### The anti-apoptotic effect of TNF-α is not mediated by production of IL-3, IL-5 or GM-CSF

Enhancement of eosinophil survival in a co-culture with mast-cells has been attributed to the production of TNF-α and GM-CSF in the culture [13]. To determine whether TNF-α-induced eosinophil survival was due to production of the known eosinophil-survival-prolonging cytokines IL-3, IL-5 or GM-CSF, TNF-α-stimulated eosinophils were cultured in the presence of relevant neutralising antibodies. These neutralising antibodies have been shown to reverse the prolonged eosinophil survival induced by addition of exogenous IL-3, IL-5 or GM-CSF in similar conditions [18]. However, neutralisation of IL-3, IL-5 or GM-CSF in the culture did not reverse TNF-α-induced inhibition of eosinophil apoptosis (Table 3).

**Table 3:** The effect of IL-3, IL-5 and GM-CSF neutralization on the antiapoptotic effect of TNF-α in human eosinophils.

		Apoptosis (% of control)	
Neutralizing antibody to	TNF-α (ng/ml)	IgG_1_ isotype control	Neutralizing antibody
IL-3	0.1	97±4	79±7
	1	76±8	72±10
	10	83±6	71±12
	100	76±13	76±12
IL-5	0.1	82±3	85±3
	1	75±1	80±11
	10	64±7	77±11
	100	65±8	77±11
GM-CSF	0.1	90±4	93±3
	1	85±3	83±5
	10	77±6	69±7
	100	65±4	66±6

Eosinophils were incubated with the IgG_1_ isotype control or the indicated neutralizing antibody to IL-3, IL-5 or GM-CSF (each at 10 µg/ml) and various concentrations of TNF-α for 40 hours. Apoptosis was assessed by measuring the relative DNA content of propidium iodide-stained cells by flow cytometry and is expressed as percentage of control, where the corresponding value obtained in the absence of TNF-α is set as 100%. Results are means ± SEM of 5–6 independent determinations using eosinophils from different donors.

### Inhibition of eosinophil apoptosis by TNF-α is mainly mediated by the TNF- receptor 1

**Figure 3:**
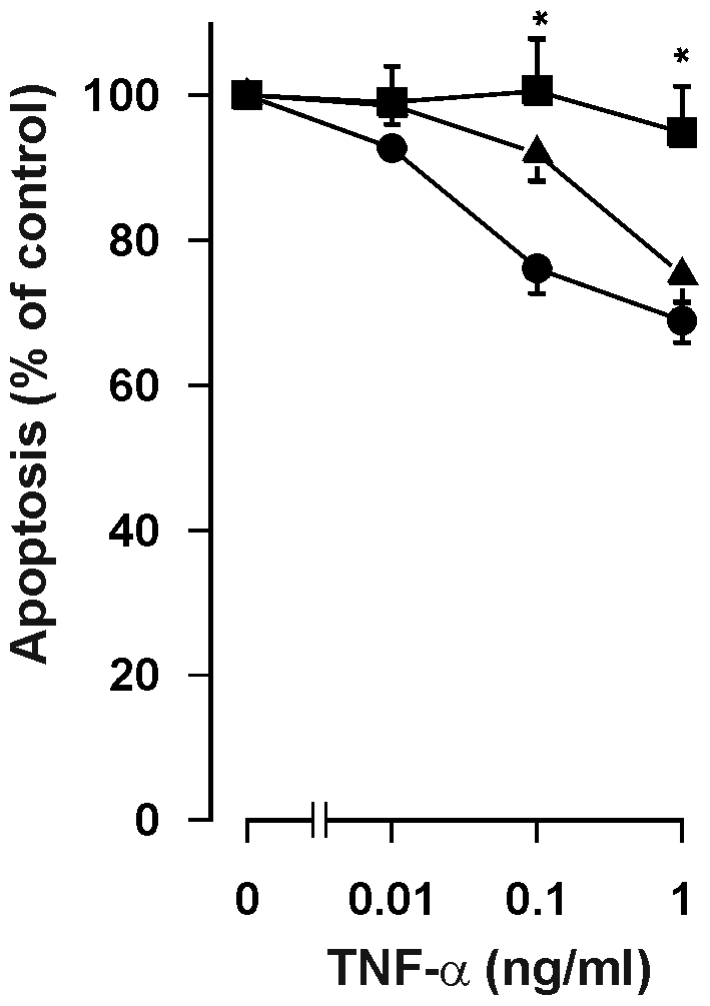
Effect of TNF-receptor antibodies on apoptosis of human eosinophils in the presence of TNF-α. The effect of TNF-R1 mAb (10 µg/ml; ▪) and TNF-R2 mAb (10 µg/ml; ▴) on the anti-apoptotic effect of TNF-α in isolated human eosinophils cultured for 40 hours. The control TNF-α concentration-response curve (•) was made in the presence of IgG_1_ isotype control (10 µg/ml). Apoptosis was assessed by flow cytometry measuring the relative DNA content of propidium iodide-stained eosinophils. Each data point represents the mean ± SEM of 6–8 independent determinations using eosinophils from different donors. Results are expressed as percentage of control. Solvent control in the absence of TNF-α is set as 100%.

Human eosinophils express both TNF receptors 1 (TNF-R1; Tnfrsf1a) and 2 (TNF-R2; Tnfrsf1b) [29]. To study which receptor subtype mediates the anti-apoptotic effect of TNF-α, eosinophils were cultured in the presence and absence of antibodies to soluble and cell surface TNF-R1 and TNF-R2. Both antibodies were used at a concentration of 10 µg/ml at which concentration there is no cross-reaction. The TNF-R1 antibody completely reversed the anti-apoptotic effect of TNF-α at concentrations of 0.1–1 ng/ml whereas the TNF-R2 antibody did not have a statistically significant effect (Fig. 3). To confirm this finding, apoptosis was analysed by Annexin-V binding assay. In the presence of IgG_1_ isotype control (10 µg/ml), TNF-α inhibited apoptosis (55.3±9.2% and 47.3±8.1% apoptotic cells in the absence and presence of 10 ng/ml TNF-α; n = 8, p<0.01). In the presence of anti-TNF-R1 antibody (10 µg/ml) TNF-α did not anymore inhibit apoptosis (55.9±10.1% and 55.0±10.7% apoptotic cells in the absence and presence of 10 ng/ml TNF-α; n = 8). In contrast, in the presence of anti-TNF-R2 antibody (10 µg/ml) TNF-α reduced the percentage of apoptotic cells (55.2±9.3% and 47.4±8.0% apoptotic cells in the absence and presence of 10 ng/ml TNF-α; n = 8, p<0.01) similarly to that seen in the presence of isotype control. Thus, the anti-apoptotic effect of TNF-α in human eosinophils seems to be mainly due to binding of TNF-α to the TNF-R1.

### Role of p38 MAP kinase and extracellular signal regulated kinase in the action of TNF-α

Previously, TNF-α has been reported to activate p38 mitogen-activated protein kinase in eosinophils [14]. To test the possibility that activation of p38 MAPK might mediate the anti-apoptotic effect of TNF-α, we used SB203580, a pharmacological inhibitor of p38 MAP kinase. SB 203580 (1 µM) did not reverse the anti-apoptotic effect of TNF-α (0.1–100 ng/ml) (Table 4). Extracellular signal regulated kinases −1 and −2 (ERK 1/2) are activated by survival-prolonging cytokines such as IL-5 and GM-CSF [3], [22], [29]. A pharmacological approach (PD98059) was used to examine the role of MAP kinase kinase (MEK) 1, an upstream activator of ERK1/2. PD 98059 (10 µM), a concentration previously shown to almost completely block IL-5-induced ERK activation in human eosinophils [22], did not affect TNF-α induced inhibition of eosinophil apoptosis (Table 4).

**Table 4:** The effect of a MEK inhibitor PD98059 and a p38 MAPK inhibitor SB203580 on the antiapoptotic effect of TNF-α in human eosinophils.

		Apoptosis (% of control)	
	TNF-α (ng/ml)	Solvent control	Inhibitor
PD98059 (10 µM)	0.1	94±8	96±3
	1	90±8	96±2
	10	84±8	84±7
	100	79±8	82±7
SB203580 (1 µM)	0.1	95±3	95±2
	1	92±4	93±2
	10	92±4	88±2
	100	89±3	80±3

Eosinophils were preincubated with the solvent control (0.5% DMSO) or the indicated inhibitor for 30 min and thereafter with various concentrations of TNF-α for 40 hours. Apoptosis was assessed by measuring the relative DNA content of propidium iodide-stained cells by flow cytometry. Results are means ± SEM of 4 (PD98059) or 10 (SB203580) independent determinations using eosinophils from different donors. The control value in the absence of TNF-α is set as 100%. The percentage of apoptotic eosinophils in the absence of TNF-α and PD98059 was 63.5±2.5 and in the presence of TNF-α (100 ng/ml) it was 50.2±5.3 (n = 4). The percentage of apoptotic eosinophils in the absence of TNF-α and SB203580 was 58.0±5.8 and in the presence of TNF-α (100 ng/ml) it was 50.9±5.1 (n = 10).

### Effect of TNF-α on IκB expression and NF-κB DNA-binding in human eosinophils

**Figure 4:**
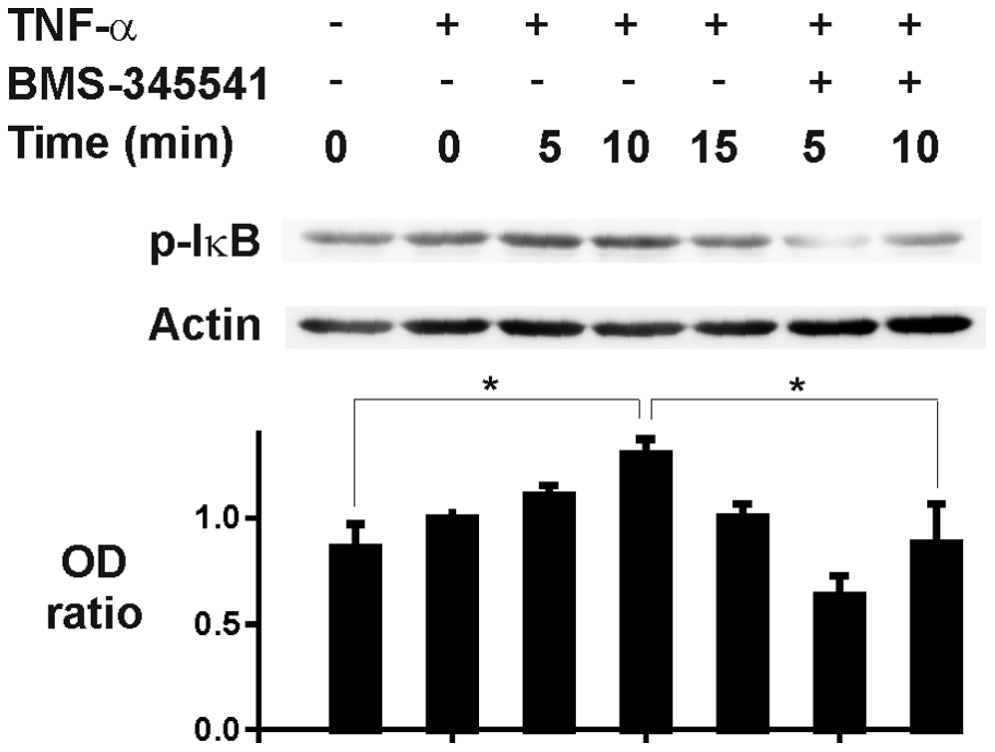
Effect of TNF-α on IκBα phosphorylation. The effect of TNF-α (100 ng/ml) in the absence and presence of IKK-1/IKK-2 inhibitor BMS-345541 (10 µM) on the phosphorylation of IκBα (p-IκB) in isolated human eosinophils. Incubations were terminated at the indicated time-points after addition of TNF-α or medium. In lower panel is shown the ratio of optical density (OD; arbitrary units) values of p-IκB and actin bands (mean ± SEM) from seven experiments using eosinophils isolated from different donors. * indicates P<0.05.

**Figure 5:**
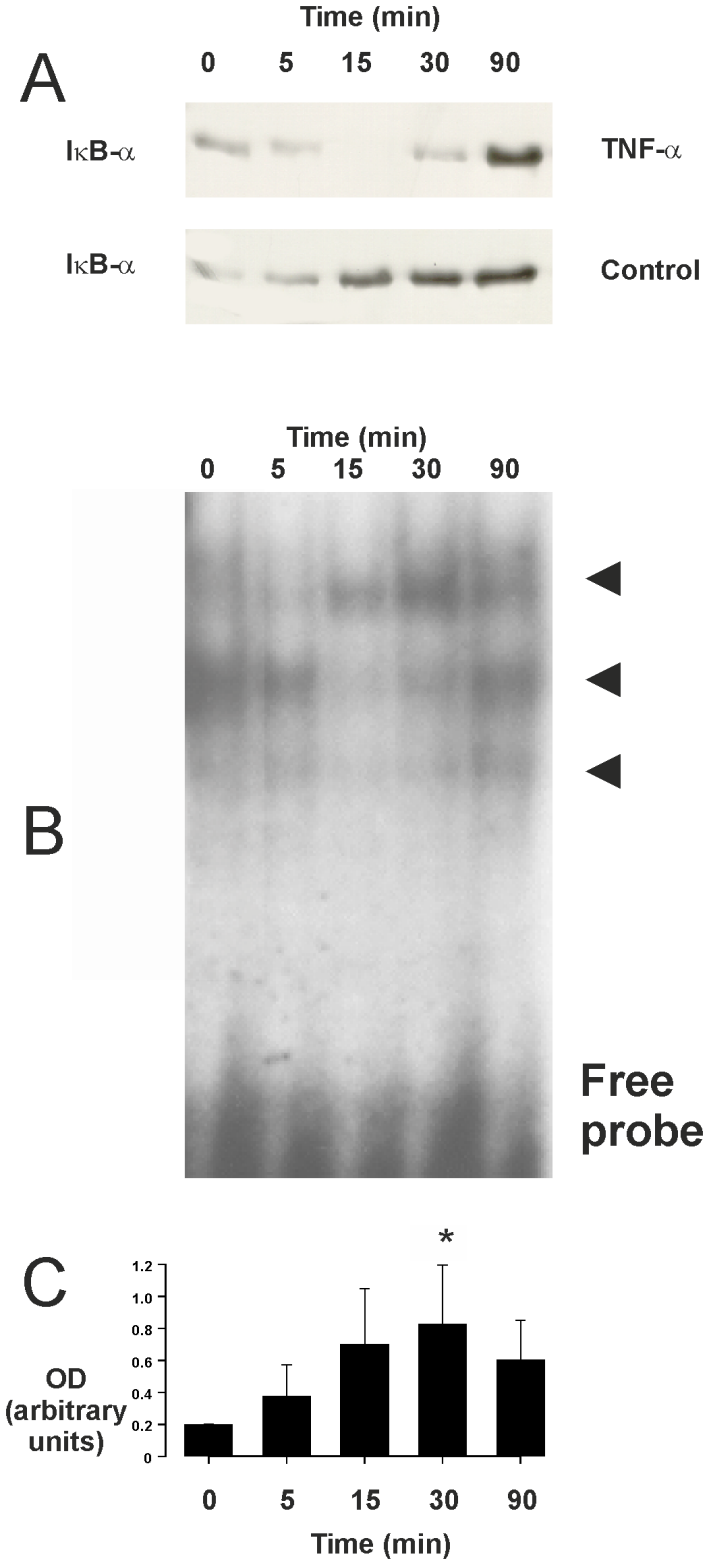
Effect of TNF-α on IκB expression and NF-κB DNA binding. In (A) is shown the effect of TNF-α (100 ng/ml) on the expression of IκBα in isolated human eosinophils. Incubations were terminated at the indicated time-points after addition of TNF-α (top panel) or medium in the simultaneously prepared control cells isolated from the same donor (lower panel). The data are representative of three separate experiments using eosinophils isolated from different donors. In (B) is shown the effect of TNF-α on NF-κB DNA binding in isolated human eosinophils. Incubations were terminated at the indicated time-points after addition of TNF-α (100 ng/ml). NF-κB DNA-binding activity was analyzed by electrophoretic mobility shift assay. Arrowheads indicate the different specific bands. In (C) is shown the total optical density (OD; arbitrary units) values of the abovementioned three specific bands (mean ± SEM) from five experiments using eosinophils isolated from different donors. * indicates P<0.05 as compared with the respective value at 0 min timepoint.

In resting cells NF-κB is localized to the cytoplasm because of binding to inhibitory protein IκB. Upon activation NF-κB-inducing kinase (NIK) is activated, which in turn activates a complex of specific IκB kinases (IKKs) resulting in IκB phosphorylation. Phosphorylation of IκB leads to a rapid ubiquitination which makes it a substrate for the proteasome [33]. Incubation of eosinophils with TNF-α for 5–15 min resulted in phosphorylation (Fig. 4) and disappearance of IκBα (Fig. 5A). Such a disappearance was not found in simultaneously prepared control cells (Fig. 5A). These results suggested that TNF-α activates NF-κB in human eosinophils. There was some NF-κB DNA binding activity in control cells as analysed by electrophoretic mobility shift assay (Fig. 5B). This DNA-binding activity was increased maximally 3-fold after incubation with TNF-α for 30 min (Fig. 5B–C). There were three separate bands (arrowheads in the figure) that were considered specific as addition of 50-fold excess of specific, but not the non-specific competitor oligonucleotide led to the disappearance of those bands (Figure S1).

### Inhibition of NF-κB reverses TNF-α-induced eosinophil survival

**Figure 6:**
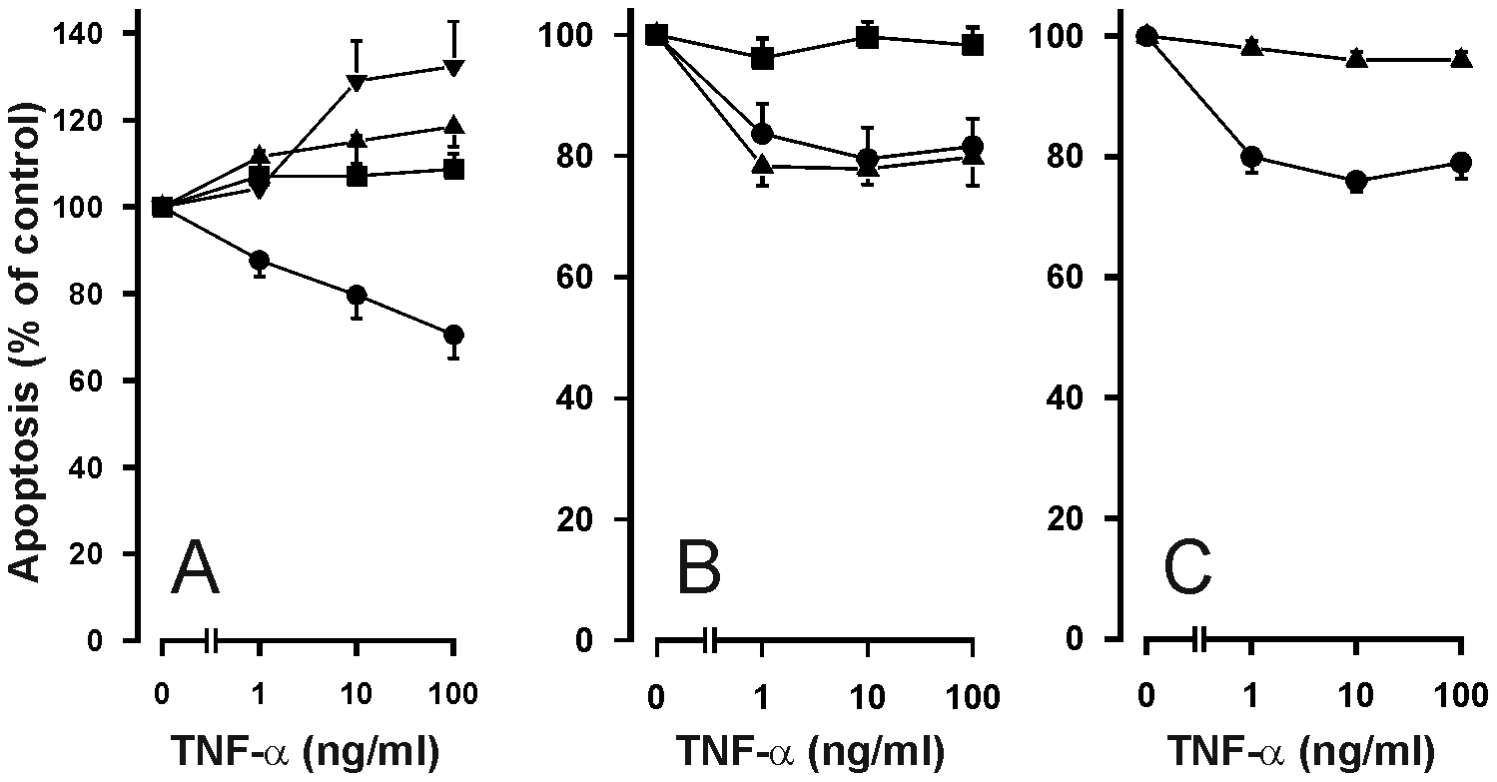
Effect of NF-κB inhibitors on apoptosis of human eosinophils in the presence of TNF-α. The effects of NF-κB inhibitors A. PDTC (▪ 1 µM; ▴ 10 µM; ▾100 µM), B. gliotoxin (▪ 0.9 µg/ml) and the inactive methylgliotoxin (▴ 0.9 µg/ml) and C. an inhibitor of IκB kinases-1 and -2, BMS-345541 (▴ 10 µM) on TNF-α-induced inhibition of apoptosis in isolated human eosinophils after 40 h culture. In each figure, the concentration-response curve of TNF-α in the absence of inhibitors (solvent control) is indicated by (•). Apoptosis was assessed by flow cytometry measuring the relative DNA content of propidium iodide-stained eosinophils. Each data point represents the mean ± SEM of 6-9 independent determinations using eosinophils from different donors. In A, the percentage of apoptotic eosinophils in the absence of TNF-α and PDTC was 57.4±5.5 and in the presence of PDTC it was 63.6±11.0 (1 µM; P>0.05), 49.1±8.0 (10 µM; P>0.05) and 33.1±2.7 (100 µM; P<0.05). In B, the percentage of apoptotic eosinophils in the absence of TNF-α and gliotoxin was 32.7±5.2 and in the presence of gliotoxin (0.9 µg/ml) it was 77.1±4.3 (P<0.001). In C, the percentage of apoptotic eosinophils in the absence of TNF-α and BMS-345541 was 53.5±2.3 and in the presence of BMS-345541 (10 µM) it was 88.2±1.6 (P<0.001).

To examine the functional role of NF-κB activation in TNF-α-driven eosinophil survival, a pharmacological approach was adopted. Eosinophil apoptosis was assessed in cultures supplemented with inhibitors of this transcription factor. Ammonium pyrroledithiocarbamate (PDTC) and gliotoxin were used as well characterised inhibitors of NF-κB [34], [35]. PDTC (Fig. 6A) and gliotoxin, but not the inactive methyl-gliotoxin (Fig. 6B) reversed the inhibitory effect of TNF-α on human eosinophil apoptosis. Interestingly, in the presence of PDTC (10 and 100 µM), TNF-α seemed to enhance eosinophil apoptosis (Fig. 6A). Phosphorylation of IκBα is critical in regulating the subsequent ubiquitination and proteolysis of IκBα, which then releases NF-κB to promote gene transcription. BMS-345541 is a selective inhibitor of IκB kinases 1 and 2 (IKK-1 and IKK-2) [36]. BMS-345541 inhibited IκBα phosphorylation (Fig. 4) and reversed the inhibitory effect of TNF-α on eosinophil apoptosis (Fig. 6C).

### The effect of TNF-α on AP-1 activation in human eosinophils

**Figure 7:**
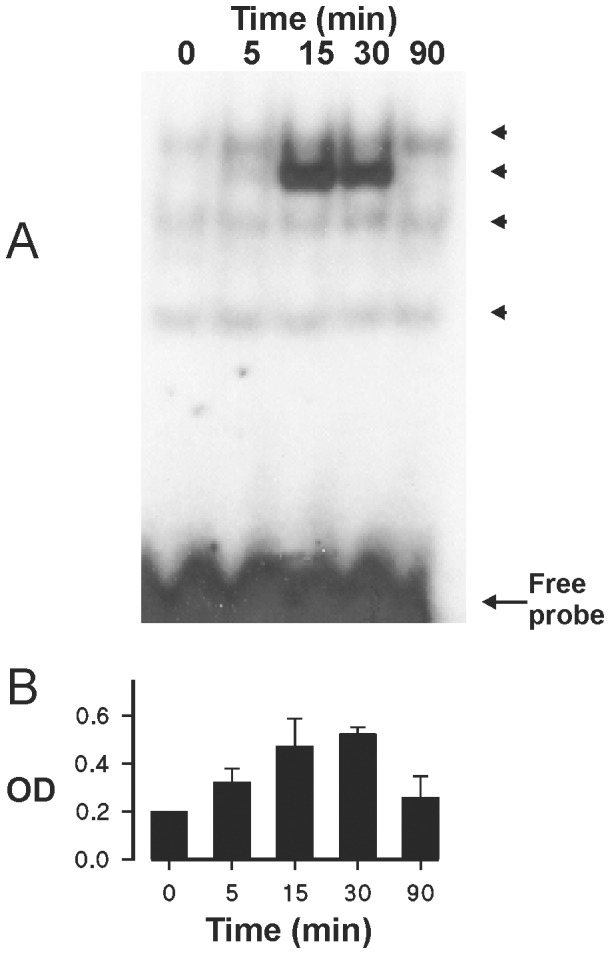
Effect of TNF-α on AP-1 activation in human eosinophils. The effect of TNF-α (100 ng/ml) on AP-1 activation in isolated human eosinophils. Incubations were terminated at the indicated time-points after addition of TNF-α. AP-1 DNA-binding activity was analyzed by electrophoretic mobility shift assay (A). Arrowheads indicate the different specific bands. The data are representative of three experiments. In (B) is shown the total optical density (OD; arbitrary units) values of the abovementioned specific bands (mean ± SEM) from 3 experiments using eosinophils isolated from different donors.

The finding that PDTC not only reverses, but may even enhance eosinophil apoptosis in TNF-α stimulated eosinophils (Fig. 6A) and that NF-κB inhibition has previously been reported to “un-mask” the ability of TNF-α to induce apoptosis in eosinophils [15], [16] suggests that other mediators might be involved. TNF-α is known to activate the redox-sensitive transcription factor AP-1 in several cell types [7]. This prompted us to test whether TNF-α is able to activate AP-1 in human eosinophils. Results demonstrate that TNF-α stimulated AP-1 DNA-binding in a time-dependent manner (Fig. 7).

**Figure 8:**
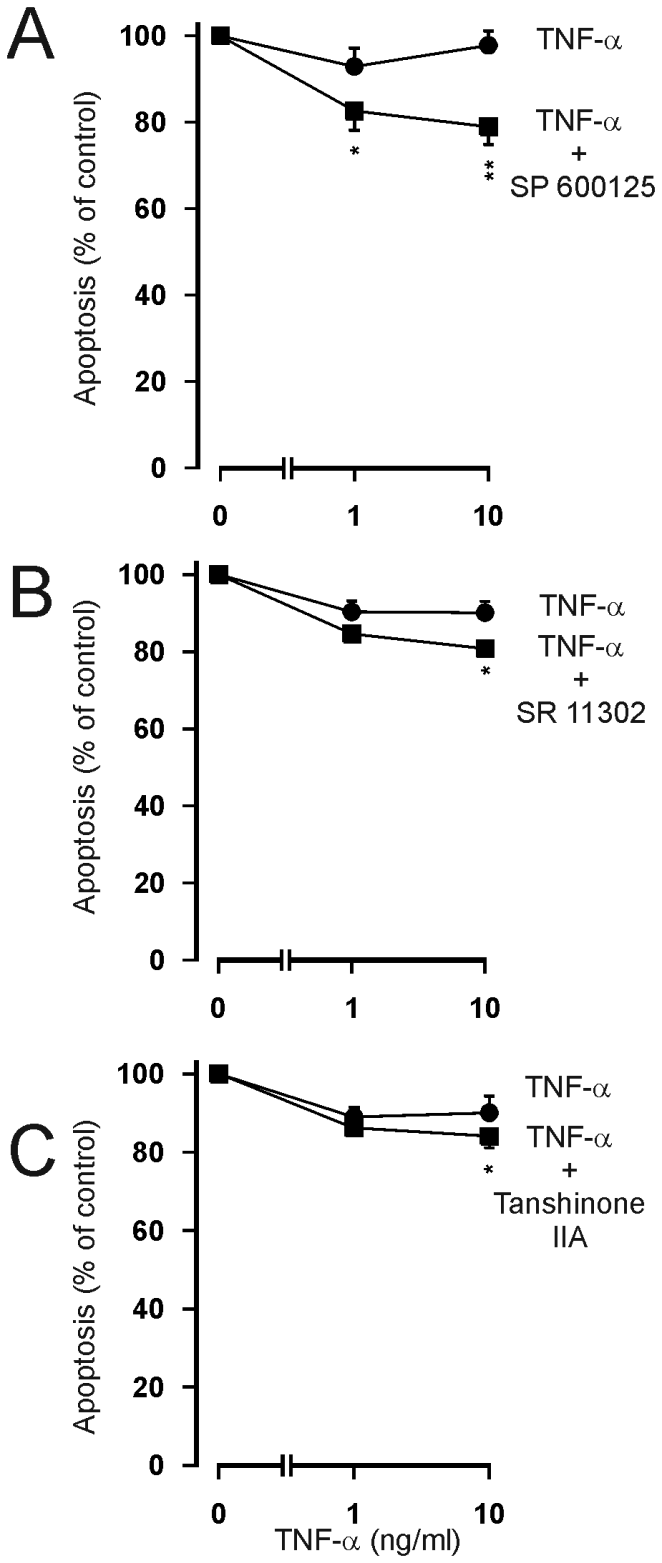
Effect of inhibitors of c-jun-N-terminal kinase and AP-1 on apoptosis of human eosinophils in the presence of TNF-α. The effects of c-jun-N-terminal kinase inhibitor SP 600125 (A: ▪ 10 µM) and inhibitors of AP-1, SR 11302 (B; ▪ 1 µM) and tanshinone IIA (C; ▪ 1 µg/ml) on TNF-α-induced inhibition of apoptosis in isolated human eosinophils after 40 h culture. In each figure, the concentration-response curve of TNF-α in the absence of inhibitors (solvent control) is indicated by (•). Apoptosis was assessed by flow cytometry measuring the relative DNA content of propidium iodide-stained eosinophils. Each data point represents the mean ± SEM of 8-14 independent determinations using eosinophils from different donors. * indicates P<0.05 and **P<0.01 as compared with the respective control in the absence of the inhibitor. In A, the percentage of apoptotic eosinophils in the absence of TNF-α and SP 600125 was 46.1±5.1 and in the presence of SP 600125 (10 µM) it was 38.4±4.8 (P<0.05). In B, the percentage of apoptotic eosinophils in the absence of TNF-α and SR 11302 was 58.6±4.9 and in the presence of SR 11302 (1 µM) it was 62.8±4.3 (P<0.05). In C, the percentage of apoptotic eosinophils in the absence of TNF-α and tanshinone IIA was 55.9±4.0 and in the presence of tanshinone IIA (1 µg/ml) it was 64.8±3.8 (P<0.001).

Activation of c-jun-N-terminal kinase (JNK) is known to precede the activation of AP-1. If AP-1 activation is “pro-apoptotic” in TNF-α stimulated cells, blocking JNK– AP-1 pathway by an inhibitor of JNK should potentiate the inhibitory effect of TNF-α on eosinophil apoptosis. In fact, SP600125, an inhibitor of JNK, amplified the inhibitory effect of TNF-α on eosinophil apoptosis (Figure 8A). To further characterize the importance of JNK-AP-1 pathway in the effects of TNF-α, two pharmacologically distinct AP-1 inhibitors, SR 11302 and tanshinone IIA [37], [38] were employed. Both SR 11302 and tanshinone IIA enhanced the survival-prolonging activity of TNF-α significantly (Fig. 8B–C).

## Discussion

In this study we have shown that in contrast to Fas, TNF-α enhanced survival of human eosinophils by inhibiting apoptosis and that the effects of TNF-α on human eosinophil survival seem to be mediated mainly via TNF-R1 and a balance between activation of NF-κB and AP-1.

In addition to the agonistic monoclonal Fas antibody, the naturally occurring agonist FasL produced an increase in the rate of eosinophil apoptosis confirming the previous reports [30]–[32] on the induction of apoptosis by cross-linking Fas by monoclonal antibodies. In contrast to Fas activation, TNF-α is a trophic factor for human eosinophils. The inhibition of eosinophil apoptosis by TNF-α was confirmed by flow cytometric analysis, DNA fragmentation assay, morphological analysis of May-Grünwald-Giemsa-stained cells and by demonstrating reduced phosphatidylserine expression on the surface of eosinophils. In several other cell types, TNF-α induces apoptotic or necrotic cell death [11], [12]. Direct induction of necrotic cell death by TNF-α could have resulted in a decreased number of apoptotic cells in the assay. However, using a test based on membrane integrity, primary necrosis as a contributing factor could be excluded. Furthermore, there were no signs of primary necrosis in the May Grünwald-Giemsa-stained cells. Thus, we conclude that TNF-α is a trophic factor for human eosinophils.

TNF-α has previously been reported to enhance eosinophil survival [17], although this has not been confirmed in other studies [15], [16]. However, the exact mode of cell death has not been analyzed in that study. The present study brings together the paucity of existing evidence that TNF-α might be a regulator of human eosinophil survival and that this may involve NF-κB. These pieces of evidence include findings that TNF-α may enhance eosinophil survival, that an inhibitor of NF-κB, gliotoxin un-masks the ability of TNF-α to induce apoptosis in eosinophils and that NF-κB is translocated to the nucleus in response to stimulation with TNF-α [14]–[17]. The data shown here convincingly demonstrates the major role of NF-κB on TNF-α-induced inhibition of eosinophil apoptosis. Thus, the present study adds to the current literature by showing several novel aspects of TNF-α signalling in human eosinophils. Firstly, in contrast to Fas-activation, TNF-α inhibits apoptosis in human eosinophils as evidenced by several different methods. Secondly, this pathway includes TNF-R1, but does not involve production of IL-3, IL-5 or GM-CSF. Thirdly, TNF-α activates NF-κB pathway in eosinophils as evidenced by phosphorylation and disappearance of IκB and by increased NF-κB DNA binding. The current results with several chemically unrelated inhibitors of NF-κB pathway affecting NF-κB activation at several steps e.g. at the level of IκB kinases (BMS-345541) or NF-κB activation itself (PDTC and gliotoxin) indicate that NF-κB activation is critical to the survival-prolonging effect of TNF-α. Fourthly, we have shown that TNF-α activates AP-1. The JNK - AP-1 pathway seems to mediate the pro-apoptotic effects of TNF-α in human eosinophils as evidenced by the amplification of the inhibitory effect of TNF-α on apoptosis during inhibition of this pathway at the level of c-jun-N-terminal kinase (SP 600125) or at the level of AP-1 itself (SR 11302, Tanshinone IIA). Furthermore, we have been able to exclude ERK1/2 and p38 MAPK as major mediators of the anti-apoptotic effects of TNF-α in human eosinophils.

Previously, both TNF-R1 and TNF-R2 have been reported to be involved in the survival-prolonging action of TNF-α in human eosinophils [17]. In the present study, the effect was mainly mediated by TNF-R1, even though we cannot completely rule out a minor role for TNF-R2. It is possible that the difference between the present results and those previously published [17] is due to the different specificity of the antibodies used.

While much has been learned about the mechanism, by which various members of the TNF superfamily signal for apoptosis, proliferation and survival, much remains unknown. For example, the reasons why TNF and its family members activate both apoptosis and anti-apoptotic pathways simultaneously and why almost all members of the TNF family induce apoptosis of some cells and proliferation of others remains to be elucidated [39]. In general, activation of caspase pathways by TNF family members have been linked to apoptosis, whereas pathways leading to activation of SODD - TRAF-2 - NF-κB pathway has been linked to anti-apoptosis, proliferation and cell survival [39], [40]. TNF-α induces also a pathway leading to activation of transcription factor AP-1, but there exists only very little information on the role of the TNF - TRAF-2 - JNK - AP-1 pathway in the regulation of apoptosis and its interaction with the NF-κB pathway. JNK/AP-1 pathway has a dual role in the regulation of cell survival as it may function as an oncogenic/antiapoptotic promoter of tumorigenesis or stress-related inducer of programmed cell death [41], [42]. In the present study, we have shown that TNF-α activates both NF-κB and AP-1. Inhibition of NF-κB pathway by PDTC even led to enhanced apoptosis by TNF-α. In the presence of a functionally active NF-κB pathway (i.e. in the absence of NF-κB inhibitors) inhibition of JNK - AP-1 pathway at different levels led to amplification of the survival-prolonging activity of TNF-α. This suggests that upon TNF-receptor ligation there exist a balance between these pathways whereby the JNK - AP-1 pathway mediates apoptosis and the NF-κB pathway prolongs eosinophil survival. As the net effect of TNF-α in the absence of any inhibitors for these pathways is stimulation of eosinophil survival, this suggests that NF-κB is more active or regulates the activity of JNK - AP-1 pathway. Interestingly, NF-κB-mediated negative feedback regulation of JNK in TNF-stimulated murine fibroblasts has been described [43], [44]. Furthermore, in the rat hepatocyte cell line RALA255-10G it was shown that the balance of NF-κB and JNK/AP-1 pathways regulates cell survival and apoptosis in response to TNF-α [45]. More recently, it has been demonstrated in human dendritic cells, that after TNF-stimulation JNK - AP-1 activity is under negative feedback control of NF-κB and can execute apoptosis. This indicates that in TNF-activated dendritic cells their apoptosis is regulated by a balance between NF-κB and JNK/AP-1 activity [46]. The present results and the literature thus suggest that in human eosinophils upon exposure to TNF-α exhibits a *yin-yang* balance between NF-κB and JNK-AP-1 pathways which determines whether the eosinophil survives or undergoes apoptosis.

Delayed eosinophil apoptosis is considered as a pathogenic mechanism in eosinophilic diseases [47] and eosinophil apoptosis is delayed in asthma [18], [19]. The results of the present study show that TNF-α is able to prolong eosinophil survival by inhibiting apoptosis. Thus, it is tempting to speculate that the up-regulated TNF-α levels in severe refractory asthma may lead to prolonged eosinophil survival and thus to increased numbers of eosinophils in the blood circulation and tissues *in vivo*. Furthermore, TNF-α not only modulates eosinophil survival but is able to activate them as well as several other cell types [7], [8], [20]. TNF-α blockers are now regarded as potential novel medications in asthma and COPD management. Recently, a trial with a TNF-α antagonist, golimumab, demonstrated that certain subgroups of asthmatics may benefit from anti-TNF-α therapies. Unfortunately, the safety profile of golimumab in patients with severe asthma was unacceptable and the study failed to demonstrate a favourable risk-benefit profile [9]. The present result that TNF-α promotes eosinophil survival and thus may contribute to eosinophilia especially in asthma or COPD exacerbations gives further support to the view [8] that anti-TNF-α therapies should not be completely abandoned and may be better targeted to the phenotype of asthmatics with high eosinophilia.

## Supporting Information

Figure S1 **Specificity of TNF-α -induced NF-κB DNA binding in human eosinophils.** Incubations were terminated at the indicated time-points after addition of TNF-α (100 ng/ml). NF-κB DNA-binding activity was analyzed by electrophoretic mobility shift assay. Nuclear extracts were incubated with 50-fold excess of either the specific unlabeled NF-κB consensus probe or with the nonspecific competitor (5′-CGC TTG AGT CAG CCG GAA-3′) prior to the addition of the labeled NF-κB probe. Arrowheads indicate the different specific bands found in each experiment.
(TIF)
