# Supplementary figures and images for: Tumour Necrosis Factor-α Regulates Human Eosinophil Apoptosis via Ligation of TNF-Receptor 1 and Balance between NF-κB and AP-1
Source: PLoS One. 2014 Feb 28;9(2):e90298. doi: 10.1371/journal.pone.0090298 (PMC3938678; doi:10.1371/journal.pone.0090298)

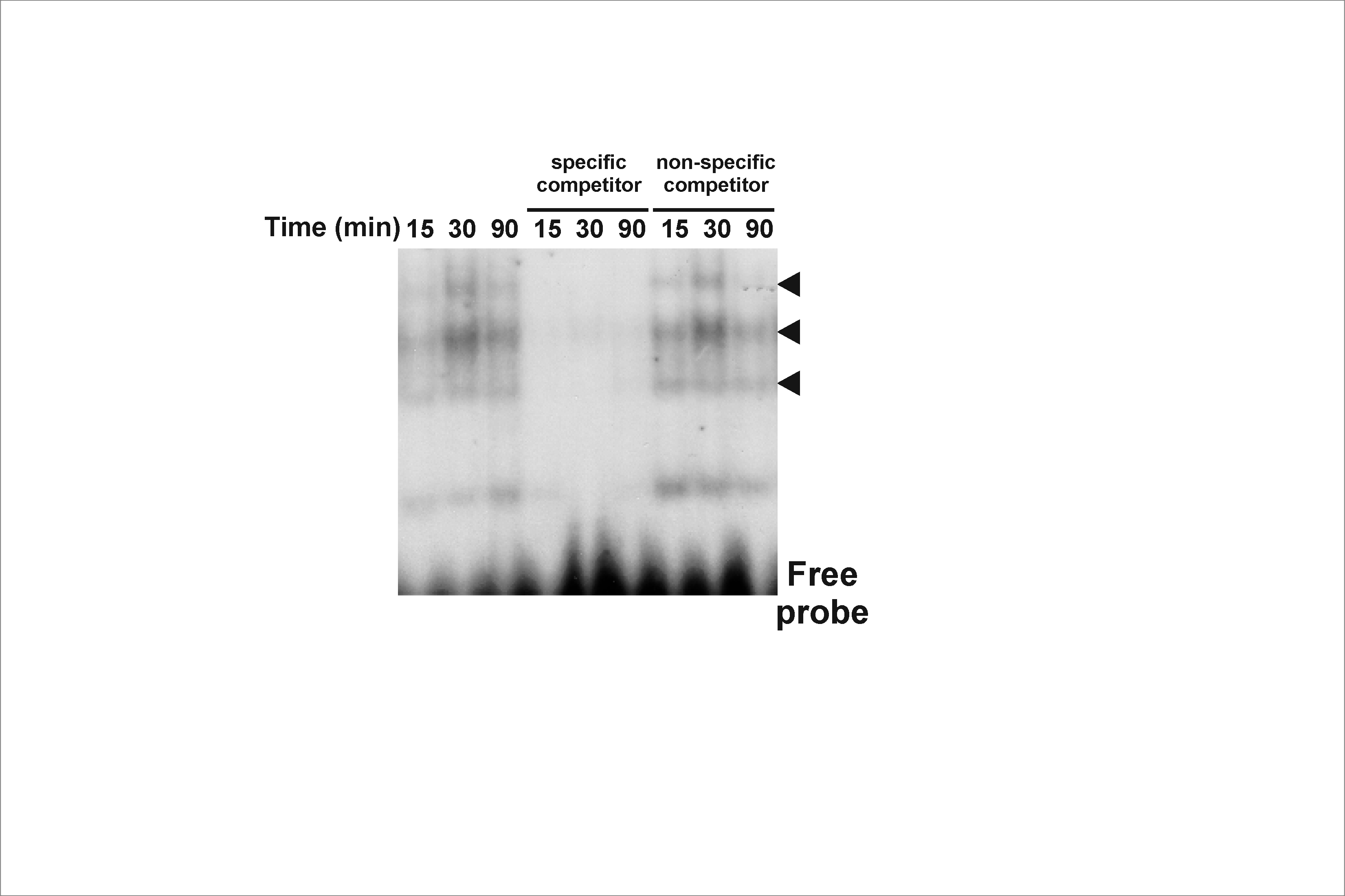

Supplement: Figure S1 — Specificity of TNF-α -induced NF-κB DNA binding in human eosinophils. Incubations were terminated at the indicated time-points after addition of TNF-α (100 ng/ml). NF-κB DNA-binding activity was analyzed by electrophoretic mobility shift assay. Nuclear extracts were incubated with 50-fold excess of either the specific unlabeled NF-κB consensus probe or with the nonspecific competitor (5′-CGC TTG AGT CAG CCG GAA-3′) prior to the addition of the labeled NF-κB probe. Arrowheads indicate the different specific bands found in each experiment. (TIF) [file pone.0090298.s001.tif]
